# Neurological Manifestations Associated With Hypomagnesemia Due to Proton-Pump Inhibitors: A Case Series

**DOI:** 10.7759/cureus.78229

**Published:** 2025-01-30

**Authors:** Lijo James, Gautham Rajan, Deepak Devarajan, Aiswarya Mohan

**Affiliations:** 1 Neurology, Caritas Hospital and Institute of Health Sciences, Kottayam, IND; 2 Nephrology, Caritas Hospital and Institute of Health Sciences, Kottayam, IND; 3 Research and Development Cell, Caritas Hospital and Institute of Health Sciences, Kottayam, IND

**Keywords:** hypocalcemia, hypomagnesemia, pantoprazole, proton pump inhibitors (ppis), seizure

## Abstract

We discuss a case series that explores the presentation and diagnostic challenges of three elderly male patients with hypomagnesemia. This series highlights the association between hypomagnesemia and hypocalcemia, both of which contribute to neurological symptoms. Proton-pump inhibitors, when taken for a long duration, are known to cause hypomagnesemia. This series also emphasizes the reversible neurological syndromes in patients with hypomagnesemia due to prolonged pantoprazole intake.

## Introduction

Proton-pump inhibitors (PPIs) are first-line therapy for most gastroesophageal acid-related disorders [1]. The class of PPI drugs includes six FDA approved medications such as rabeprazole, lansoprazole, pantoprazole, esomeprazole, omeprazole, and dexlansoprazole. They are the most effective drugs currently available for suppressing gastric acid secretion. They are particularly effective drugs for treating gastric and duodenal ulcers, gastroesophageal reflux disease, and erosive esophagitis [2]. They inhibit the release of gastric acid from parietal cells by blocking hydrogen potassium adenosine triphosphatase (H^+^/K^+ ^ATPase), also known as proton pump. In addition to blocking the H^+^/K^+^ ATPase, they also block vacuolar ATPases at various sites, including brain microglial cells [3]. Notably, PPIs have been observed to cross the blood-brain barrier.

PPIs are generally well-tolerated drugs. PPI-induced hypomagnesemia is a rare but serious adverse effect of this widely prescribed medication [4]. PPIs, which are often used to treat acid reflux symptoms, can lead to hypomagnesemia and hypocalcemia with prolonged use. PPI-induced hypomagnesemia has been recognized since 2006 [5].

Hypomagnesemia has recently been recognized as a side effect of PPIs. It is defined as serum magnesium levels less than 1.46 mg/dL, and it can cause several kinds of symptoms ranging from mild gastrointestinal symptoms to significant conditions such as cardiac arrhythmias and neurological complications [6]. PPIs are proposed to decrease the activity of transient receptor potential melastatin 6 (TRPM6), which causes reduced magnesium absorption from the intestine [7]. Low serum magnesium levels can result in serious adverse events like neurological problems such as seizures [8].

PPI-induced neurological symptoms like seizures, tremors, and dementia in the elderly represent a unique diagnostic and therapeutic challenge [9-11]. Elderly patients with seizures often present with subtle or atypical manifestations, such as confusion or motor symptoms, which can be easily misattributed to other conditions like stroke, dementia, or infections [12].

In this case series, we aim to highlight the importance of assessing for all possible electrolyte abnormalities in elderly patients presented with neurological symptoms, taking a comprehensive drug history which involves all undocumented over-the-counter (OTC) medications, and patient education in preventing further episodes.

## Case presentation

Case 1

A 57-year-old male was referred to the neurology department with sudden seizure episodes. The patient had no previous history of epilepsy but reported a three-month history of appetite loss, fever for 20 days, and nausea for three days. His medical history included coronary artery disease (CAD) treated with percutaneous coronary intervention (PTCA) following an inferior wall myocardial infarction (IWMI), and benign paroxysmal positional vertigo (BPPV).

On examination, the patient was drowsy but able to move with normal muscle power (5/5). His blood pressure was 130/90 mmHg. Laboratory investigations revealed significant hypocalcemia (7.7 mg/dL) and hypomagnesemia (0.42 mg/dL), alongside mild hyponatremia (132 mEq/L) and hyperglycemia (random blood sugar: 206 mg/dL), suggesting newly diagnosed diabetes mellitus (Table 1). His electrolyte disturbances were likely worsened by chronic use of PPIs, specifically pantoprazole (40 mg daily) for over 10 years. His troponin I was negative.

**Table 1:** The Laboratory Values of All Three Cases

	Value	Reference Range
Case 1		
Magnesium	0.42	1.8-2.6 mg/dL
Serum Calcium	7.7	8.4-10.2 mg/dL
Haemoglobin	10.3	13-16.5 g/dL
Blood Urea	14	17-49 mg/dL
Serum Sodium	132	136-145 mEq/L
Case 2		
Magnesium	0.4	1.8-2.6 mg/dL
Serum Calcium	7.7	8.4-10.2 mg/dL
Haemoglobin	13.0	13-16.5 g/dL
Blood Urea	12	17-49 mg/dL
Serum Sodium	117	136-145 mEq/L
Case 3		
Magnesium	0.95	1.8-2.6 mg/dL
Serum Calcium	11.1	8.4-10.2 mg/dL
Haemoglobin	10.3	13-16.5 g/dL
Blood Urea	12	17-49 mg/dL
Serum Sodium	125	136-145 mEq/L

The seizure was attributed to severe hypomagnesemia and hypocalcemia which were corrected during his hospital stay. His diabetes mellitus was also managed with oral hypoglycemic, and his CAD was stabilized with antiplatelet therapy and statins. He responded well to treatment and was discharged with advice on dietary calcium and magnesium supplementation, alongside follow-up for diabetes and cardiac management. He was advised to stop PPI and to take ranitidine (H2 blocker).

Case 2

A 68-year-old male presented with a gradual onset of difficulty walking, slowness in daily activities, and bilateral hand numbness. His Glasgow Coma Scale (GCS) score was 14 (E4 M6 V4), as reported by his parent. He had a history of diabetes mellitus, hypertension, dyslipidemia, osteoarthritis, and a prior cerebrovascular accident (left occipital lobe infarct). On general examination, he was conscious and oriented, with slow saccadic eye movements, short steps, and a slow gait. The clinical picture was suggestive of Parkinsonism. His blood pressure was 154/100 mm of Hg. Laboratory investigations revealed significant hypocalcemia (7.7 mg/dL) and hypomagnesemia (0.4 mg/dL) (Table 1). He showed significant electrolyte disturbances, including hyponatremia, likely due to thiazide diuretics. Initially, hypomagnesemia was thought to be due to thiazide diuretics, leading to its discontinuation. Despite starting Syndopa, his symptoms persisted. Follow-up at three to six months revealed persistent hypomagnesemia.

Despite initial improvement, the cause of his Parkinsonism remained unclear. After further investigation, including ruling out other potential causes, his chronic use of pantoprazole for over 10 years raised suspicion to be the contributing factor for hypomagnesemia and Parkinsonism. Subsequent discontinuation of pantoprazole led to significant improvement in his Parkinsonism-like symptoms, confirming a diagnosis of PPI-induced Parkinsonism secondary to hypomagnesemia [13]. Serial monitoring of serum magnesium levels was done and the patient’s condition stabilized. After the discontinuation of pantaprazole, his electrolytes normalized, and his neurological symptoms improved and we could stop his anti-Parkinsonism medication.

Case 3

A 70-year-old male presented to the department with jerky movements of his left focal seizure. He had a history of diabetes mellitus and bilateral grade IV osteoarthritis of the knees. One week prior to admission, he had experienced a head injury after being struck by a falling coconut; however, a CT scan of the head was normal.

On examination, he was drowsy and disoriented but muscle power in all limbs was preserved. His admission blood pressure was elevated at 170/100 mmHg. Laboratory investigations revealed significant hypomagnesemia (0.95 mg/dL) and hyponatremia (125 mEq/L), with random blood sugar elevated at 202 mg/dL (Table 1). The hypomagnesemia was suspected to be a contributing factor to his seizures as all other likely contributing factors were ruled out. Upon detailed history taking, it was discovered that the patient had been using pantoprazole OTC for more than 10 years.

During his hospital stay, his electrolyte imbalances were corrected with magnesium and calcium supplements, and pantoprazole was discontinued. Subsequently, his seizures were controlled with antiepileptic medication. His diabetes was managed with oral hypoglycemic, and he was advised to undergo further evaluation for his osteoarthritis. The patient's condition was stable and electrolytes were back to normal level. The patient was discharged with discharge medications.

## Discussion

PPIs have been used in clinical practice for about 30 years to treat patients of all ages. All currently approved PPIs are benzimidazole derivatives [10]. Chronic use of high-dose PPIs appears to affect calcium, magnesium, and vitamin B12 absorption because acid promotes digestion and ionization of less soluble forms of dietary calcium, as well as the release of food-bound vitamin B12 [7,10]. More recently, it has been reported that hypomagnesemia may also be induced by PPIs. The association between symptomatic hypomagnesemia and PPI use was first described in two patients in 2006 [6,8,14,12].

Magnesium is an essential ion that is important in many metabolic pathways. It is also the fourth most abundant cation in the body. Approximately 99% of the magnesium in the body is found within cells, with bone accounting for 50% to 65% of the total [13]. In our patients, marginally low levels of hypomagnesemia and hypocalcemia were noted.

Even though hypomagnesemia is defined as magnesium levels below 0.7 mmol/L, clinical signs typically appear when magnesium levels fall below 0.5 mmol/L in our case. These include reversible neurological symptoms [15]. Hypomagnesemia is primarily caused by taking PPIs for an extended period, but it has also been found in critically ill patients after a brief course of PPIs [16]. As evident in the patients described, magnesium levels were markedly low (ranging from 0.4 to 0.95 mg/dL), contributing to the seizures. The long-term use of PPIs causes reduced magnesium absorption from the intestine. Notably, pantoprazole, a PPI, was implicated in the prolonged use of all three patients, emphasising its potential adverse effects when used chronically. Hypomagnesemia is the electrolyte imbalance that raises the greatest concern when prescribing PPIs [17].

In our patients along with hypomagnesemia, hypocalcemia was also a potential consequence of PPI therapy and was evident in all of the cases [2,12,18-20]. The concurrent electrolyte imbalances, such as hyponatremia in all three cases, further compounded the risk of developing seizures. Patients who continue to take PPIs for a long period experience decreased gastric acid secretion and lowered vitamin B-12 absorption [20]. The administration of magnesium and calcium supplements proved effective in resolving the reversible neurological disorders, contributing to a marked improvement in the patient's condition. This management approach corrected the underlying metabolic imbalance, supporting overall recovery [3,14,20].To the best of our knowledge, this is the first case series from South India which showed PPI-induced hypomagnesemia presenting with neurological symptoms in elderly patients.

There are a few limitations to this study. Firstly, the small sample size of only three patients limits the generalizability of the findings, as a larger cohort is needed to validate the observed association between prolonged PPI use and hypomagnesemia-related neurological manifestations. Secondly, the lack of a control group makes it difficult to establish a direct causal relationship between PPI use and the symptoms, as other factors such as underlying comorbidities or concurrent medications could also contribute to the findings. Additionally, the retrospective nature of the study relies on patient history and available medical records, which may introduce recall bias or missing data, particularly regarding the exact duration and dosage of PPI use.

## Conclusions

This unique case series demonstrates that patients with prolonged use of PPI intake can develop hypomagnesemia which can manifest with reversible neurological problems such as encephalopathy, Parkinsonism, and seizure. In an elderly patient, if there are unexplained symptoms of neurological illness such as seizures, encephalopathy or signs of Parkinsonism and if he is reported to be having a long-term history of PPI, PPI-induced hypomagnesemia should be suspected as a differential diagnosis. Additionally, it is critical to assess the causes of hypomagnesemia, when seizure occurs with hyponatremia with serum sodium levels between 120 and 130 mEq/L for additional causes like hypomagnesemia. It is recommended to stop PPIs if they are not indicated by default prescription and to maintain a high suspicion of PPI-induced hypomagnesemia in patients using PPIs for extended periods.
